# Neural circuit mapping of waiting impulsivity and proactive inhibition with convergent evidence from fMRI and TMS

**DOI:** 10.1192/j.eurpsy.2023.2088

**Published:** 2023-07-19

**Authors:** N. Skandali, K. Baek, S. Sallie, S. Sonkusare, A. Mandali, V. Ritou, V. Caesaro, V. Caesaro, V. Voon

**Affiliations:** 1 Psychiatry, University of Cambridge; 2 Liaison Psychiatry, Addenbrooke’s hospital, Cambridgeshire and Peterborough NHS Foundation Trust, Cambridge, United Kingdom

## Abstract

**Introduction:**

Waiting and stopping are essential and distinct elements of appropriate behavioral control with its dysfunction implicated in various impulsivity related mental disorders. Although rodent and human studies have investigated both phenomena, the role of preparing to stop in waiting impulsivity has rarely been investigated. Furthermore, convergent evidence from multi-modal investigation tools remains a poorly utilized approach in addressing such questions.

**Objectives:**

Here, we conducted two separate, but hierarchical studies, using functional magnetic resonance imaging (fMRI) to map the neural circuit involved in waiting impulsivity and proactive stopping, and subsequently provide mechanistic and causal evidence of disruption of this circuit by transcranial magnetic stimulation (TMS). In the second study, based on our fMRI study data, we attempted to investigate possible causation between the LIFG and waiting impulsivity by modulating LIFG, i.e. non-invasively producing a “virtual lesion” with an inhibitory transcranial magnetic stimulation (TMS) protocol called continuous theta burst stimulation

**Methods:**

We recruited 41 healthy volunteers who performed an adapted version (1CSRT) of the well-established 5 choice serial reaction time task to capture waiting impulsivity. We developed a novel task measuring proactive inhibition. We scanned participants while completing these two tasks. Our fMRI data showed a strong association between LIFG activity and waiting impulsivity on the 1CSRT task. We conducted a single-blind, randomized, between-subjects design of cTBS of the LIFG on a sample of 51 healthy volunteers who completed an adapted version of the 1CSRT task (2CSRT task). Our a priori hypothesis was that cTBS would transiently decrease local cortical activity of the LIFG and increase the frequency of premature responses on both fixed and delayed cue-target interval trials on the 2CSRT task.

**Results:**

We first show a shared neural network comprising the pre-supplementary motor area and bilateral anterior insula underlying both waiting impulsivity and proactive stopping. We further demonstrate activity in dorsomedial prefrontal cortex and left inferior frontal gyrus (LIFG) negatively correlated with waiting impulsivity in trials with additional target onset delay. We demonstrate active stimulation significantly increased waiting impulsivity.

**Conclusions:**

In these two studies, we validated a novel task measuring proactive inhibition. We further validated the significance of task structure for assessing distinct aspects of impulsivity, which is of translational interest. We further established a causal role of lIFG for waiting impulsivity thus highlighting the integrity of LIFG and related neural circuitry required in waiting impulsivity.

**Disclosure of Interest:**

None Declared

